# Comprehensive molecular characterizations of stage I–III lung adenocarcinoma with tumor spread through air spaces

**DOI:** 10.3389/fgene.2023.1101443

**Published:** 2023-02-02

**Authors:** Ronghao Ye, Yongfeng Yu, Ruiying Zhao, Yuchen Han, Shun Lu

**Affiliations:** ^1^ Shanghai Lung Cancer Center, Shanghai Chest Hospital, Shanghai Jiao Tong University, Shanghai, China; ^2^ Department of Pathology, Shanghai Chest Hospital, Shanghai Jiao Tong University, Shanghai, China

**Keywords:** lung cancer, adenocarcinoma, spread through air spaces (STAS), next-generation sequencing (NGS), disease-free survival (DFS)

## Abstract

**Purpose:** The aim of this study is to investigate integrative genomic spectra of stage I–III lung adenocarcinoma with tumor spread through air spaces (STAS).

**Methods:** We retrospectively identified 442 surgically resected lung adenocarcinoma patients of pathological stage I–III in Shanghai Chest Hospital from January 2018 to February 2021. Surgically resected tissues were used for next-generation sequencing (NGS) with a panel of 68 lung cancer‐related genes to profile comprehensive molecular characterizations.

**Results:** A total of 442 cases were analyzed, including 221 (50%) STAS-positive (SP) and 221 (50%) STAS-negative (SN) lung adenocarcinoma patients. In total, 440 cases (99.6%) were positive for the overall mutational spectrum, and the higher mutational genes were EGFR, TP53, KRAS, ALK, SMAD4, and ERBB2 (62%, 42%, 14%, 10%, 7%, and 7%, respectively). Compared with the SN population, there was significantly lower EGFR alteration in the single-nucleotide variant (SNV) mutation spectrum (52.5% vs 69.7%, *p* < 0.001) and significantly higher TP53 alteration in the SP population (49.8% vs 34.8%, *p* = 0.002). EGFR L858R missense mutation (19.5% vs 37.6%, *p* < 0.001) and ERBB2 exon 20 indel mutation (1.8% vs 5.9%, *p* = 0.045) were more frequent in the SN population. The detection rate of ALK fusion rearrangements in the SP population was significantly higher than that in the SN population (13.1% vs 2.3%, *p* < 0.001). In the analysis of signaling pathways, no significant difference was discovered between SP and SN patients. No difference in 1-year disease-free survival was observed between SP and SN patients in this study.

**Conclusion:** Significant differences exist in stage I–III lung adenocarcinoma patients with STAS in molecular characterizations.

## Introduction

Lung cancer, a common solid tumor, seriously threatens people’s mental and psychological health. It was demonstrated in the 2020 WHO International Agency for Research on Cancer (IARC) global cancer data report that the incidence of lung cancer is approximately 2.2 million per year and mortality is approximately 1.8 million per year (Kordiak et al., 2022). Notably, non-small-cell lung cancer (NSCLC) accounts for 85% of all lung cancers, and one of the major pathological subtypes of NSCLC is lung adenocarcinoma (Yang et al., 2020). Despite the development of imaging techniques that can detect tumors at an early stage, lung adenocarcinoma is associated with a mounting mortality rate due to the poor prognosis and high recurrence rate, which could be explained by tumor invasion and metastasis. Spread through air spaces (STAS), a recently discovered form of infiltrates raised by the WHO in 2015, has already received much attention in the field of lung cancer (Ma et al., 2019).

STAS is defined as tumor cell propagation through the alveolar space into the lung tissue beyond the margins of the tumor. Tumor cells usually form microbial structures, solid tumor cell islands, or the spread of single tumor cells (Han et al., 2021). It has been estimated that approximately 15%–60% of lung cancer patients are STAS-positive (SP) (Eguchi et al., 2019; Tian et al., 2021), and the SP population is more frequently seen in late-stage lung cancer patients. Moreover, SP lung cancer patients are associated with low recurrence-free survival and overall survival (Toyokawa et al., 2018a; Han et al., 2021; Ikeda et al., 2021). Kadota et al. (2015) discovered that the cumulative recurrence rate of distal and local recurrence in SP patients was significantly higher than that in STAS-negative (SN) patients. Several retrospective studies have found that STAS can occur in nearly all pathological types of lung cancer (Lu et al., 2017; Yokoyama et al., 2018; Aly et al., 2019). Among them, STAS is most frequently observed in lung adenocarcinoma (ADC). It has been demonstrated that STAS is an independent prognostic factor for poor tumor-related outcomes and is associated with early-stage ADC sublobar resection (LR, wedge-shaped pulmonary resection, and segmentectomy) (Kadota et al., 2015; Toyokawa et al., 2018b; Shiono et al., 2018).

Currently, the correlation between STAS and the molecular characteristics of lung cancer is rarely reported, and thus its mechanism is still unclear (Ikeda et al., 2021). A few studies have shown high expression of vimentin (Upton et al., 1986; Karim et al., 2017; Jia et al., 2020) and low expression of E-cadherin (Jia et al., 2020) in SP patients. This indicates a potential relationship between STAS and the biomarkers of epithelial–mesenchymal transition (EMT), which is important in tumor cell migration and invasion. Liu et al. (2018) found that the elevated expression of metastasis-related protein 1 (MTA1) is correlated with STAS, which indicated the important effect of STAS on lung cancer metastasis and poor prognosis. In addition, some studies have shown that STAS occurs more frequently in tumors with ROS1 and ALK rearrangements (Kadota et al., 2019; Tian et al., 2021; Pyo and Kim, 2022). However, interestingly, some high-profile mutations, including BRAF, EGFR, and KRAS, have not been definitively linked to STAS status as they have presented mixed results in several studies (Toyokawa et al., 2018b; Jia et al., 2020). Other genes (HER2, PD-L1, TTF1, Napsin, and CK7) were also investigated, but no statistical significance was presented (Warth et al., 2015; Toyokawa et al., 2018b; Toyokawa et al., 2018c; Hu et al., 2018).

In summary, the pathogenesis of STAS is incompletely investigated, and further molecular biological characterization studies are urgently needed to update current understanding. In this study, next-generation sequencing (NGS) was conducted on the pathological tissues of SP and SN lung adenocarcinoma patients grouped by propensity score matching (PSM), and 68 genes related to lung cancer targeted therapies were analyzed to reveal the mutation spectrum and biological associations with STAS.

## Methods

### Data collection

The study protocol was evaluated and approved by the Shanghai Chest Hospital Institutional Review Board. Between 1 January 2018 and 28 February 2021, we identified patients who underwent surgical resection and tested for 68 targeted lung cancer therapy-related genes. The clinical characteristics of patients, including age, gender, smoking history, P-stage, and type of surgery, were searched and collected from the original medical records. Based on the eighth edition of the AJCC/UICC staging system, the P-stage was restaged (Jia et al., 2020). Follow-ups ranged from 1 to 12 months. There were 240 SP lung cancer patients, including two large cell carcinomas, five squamous cell carcinomas, six small cell carcinomas, and 227 lung adenocarcinomas. The analysis was narrowed to ADC subjects due to insufficient cases of other pathological types of lung cancer except for ADC. A total of 227 ADC patients consisted of 97 stage I subjects, 43 stage II subjects, 81 stage III subjects, and six stage IV subjects. Owing to the small sample size of stage IV ADC and palliative surgery conducted in these patients, a total of 221 SP ADC patients (stage I: 97, stage II: 43, and stage III: 81) and 2027 SN patients (stage I: 1467, stage II: 288, and stage III: 272) were included. Propensity score matching (PSM) (match ratio 1:1 and caliper value 0.03) was used to exclude other confounding factors, such as STAS status, including tumor stage, surgical mode, gender, age, and smoking history. The SP population was matched with the SN population (stage I: 97, stage II: 43, and stage III: 81). Tumor tissue samples from patients were obtained and reviewed by two qualified pathologists independently. Any disagreement was resolved by discussion. The presence or absence of STAS was reported following the unanimous conclusion of the two pathologists. Cases were classified based on whether STAS was present or not. DNA was extracted for the targeted sequencing of 68 genes. For both SP and SN groups, genetic mutation profiles and biological associations were analyzed.

### Targeted tumor next-generation sequencing and analysis

Formalin-fixed paraffin-embedded (FFPE) sections of surgically resected tissues were collected for DNA extraction. The tissue DNA was extracted using a QIAamp DNA FFPE Tissue Kit (Qiagen, Hilden, Germany) and subsequently sheared with Covaris M220 (Covaris, MA, United States) for end-repair, phosphorylation, and adapter connection. DNA quality and size were measured using a Qubit 2.0 Fluorometer and double-stranded DNA high-sensitivity detection kit (Life Technologies, Carlsbad, CA, United States). No less than 50 ng of tissue DNA was applied for NGS library construction and target capture using a panel of 68 lung cancer-related genes (spanning 245 kb of the human genome) (Lung Core, Burning Rock Biotech, Guangzhou, China) (Supplementary Table S1). Indexed samples were paired-end sequenced using a NextSeq500 sequencer (Illumina, Inc., Madison, WI, United States) with 1,000-fold paired-end reads and target sequencing depth.

Sequence data were mapped to the reference human genome (hg19) using the Burrows–Wheeler Aligner v.0.7.10 platform (Li and Durbin, 2009). Genome Analysis Toolkit v.3.2 (McKenna et al., 2010) and VarScan (Koboldt et al., 2009) were applied for local alignment optimization, variant calling, and annotation. Sites with depths below 100 were excluded. Indels require at least five backward reads and SNV calls require at least eight backward reads. Variants with a population frequency greater than 0.1% were classified as SNPs based on the ExAC 1,000 Genomes, dbSNP, and ESP6500SI-V2 databases and were not further analyzed. ANNOVAR (Wang et al., 2010) and SnpEff v3.6 (Yen et al., 2017) were used for the remaining variants. DNA translocation analyses were conducted using TopHat2 (Falch et al., 2018) and FACTERA 1.4.3 (Newman et al., 2014).

### Statistical analysis

All statistical analyses were conducted using R software (version 4.0.3, , Austria). Propensity score matching (PSM) was used to control for the confounding effects of the clinical parameters. Propensity scores for all lung adenocarcinoma patients were calculated using multivariate logistic regression with the following covariates: age, sex, smoking history, TNM stage, and surgery protocol. In the matched cohort, 221 SP patients were matched 1:1 with 221 SN patients. The intergroup clinical features were compared using the chi-squared test.

All mutation detection rate differences and mutually exclusive concomitant analyses between STAS+/STAS- are based on Fisher’s exact test. Genes were listed when *p* < 0.05 and *p* < 0.01. Genomic maps were clustered using the R package NMF (version 0.23.0) using a non-negative matrix factorization algorithm.

The primary outcome of clinical follow-ups was designed as disease-free survival (DFS), which was defined as the period from surgery to recurrence/cancer-related death. The DFS curves were drawn using Kaplan–Meier analysis with a log-rank test. Moreover, the hazard ratio (HR) and 95% confidence interval (CI) were calculated using Cox proportional hazard regression analysis for examining clinical characteristics. Statistical significance was set at *p* < 0.05.

## Results

### Baseline clinical characteristics

PSM was performed separately for age, gender, smoking history, TNM stage, and surgery type to ensure an exact balance between SP and SN patients in the matched cohort. A total of 442 patients were enrolled, including 221 (50%) SP patients and 221 (50%) SN patients, with 49.8% (220/442) males and 50.2% (222/442) females. In total, 58.4% (258/442) of the subjects were aged over 60 years. The vast majority of patients were non-smokers, accounting for 84.6% (374/442), and smoking patients accounted for 15.4% (68/442). Patients undergoing pneumonectomy or lobectomy accounted for 80.5% (356/442) and segmental or wedge resection accounted for 19.5% (86/442). In the cohort, 43.9% (194/442) were diagnosed with stage I, 19.5% (86/442) with stage II, and 36.7% (162/442) with stage III. The baseline characteristics before and after PSM are shown in Table 1.

**TABLE 1:** Clinical characteristic baseline before and after PSM.

Characteristic	Entire cohort (n = 2248)			Matched cohort (n = 442)		
	STAS-negative (%)	STAS-positive (%)	*p*-value	STAS-negative (%)	STAS-positive (%)	*p*-value
Gender			0.009			1
Female	1,209 (59.6)	111 (50.2)		111 (50.2)	111 (50.2)	
Male	818 (40.4)	110 (49.8)		110 (49.8)	110 (49.8)	
Age			0.017			0.846
≤60	1,021 (50.4)	92 (41.6)		88 (39.8)	91 (41.2)	
>60	1,006 (49.6)	129 (58.4)		133 (60.2)	130 (58.8)	
Smoking status			<0.001			0.896
Non-smoker	1851 (91.3)	185 (83.7)		187 (84.6)	185 (83.7)	
Smoker	176 (8.7)	36 (16.3)		34 (15.4)	36 (16.3)	
Resection			<0.001			1
Pneumonectomy/lobectomy	1,158 (57.1)	178 (80.5)		178 (80.5)	178 (80.5)	
Segmental/wedge	869 (42.9)	43 (19.5)		43 (19.5)	43 (19.5)	
Stage			<0.001			1
I	1,467 (72.4)	97 (43.9)		97 (43.9)	97 (43.9)	
II	288 (14.2)	43 (19.5)		43 (19.5)	43 (19.5)	
III	272 (13.4)	81 (36.7)		81 (36.7)	81 (36.7)	

PSM, propensity score matching; STAS, spread through air spaces.

### Characterization of genetic alterations

**FIGURE 1:**
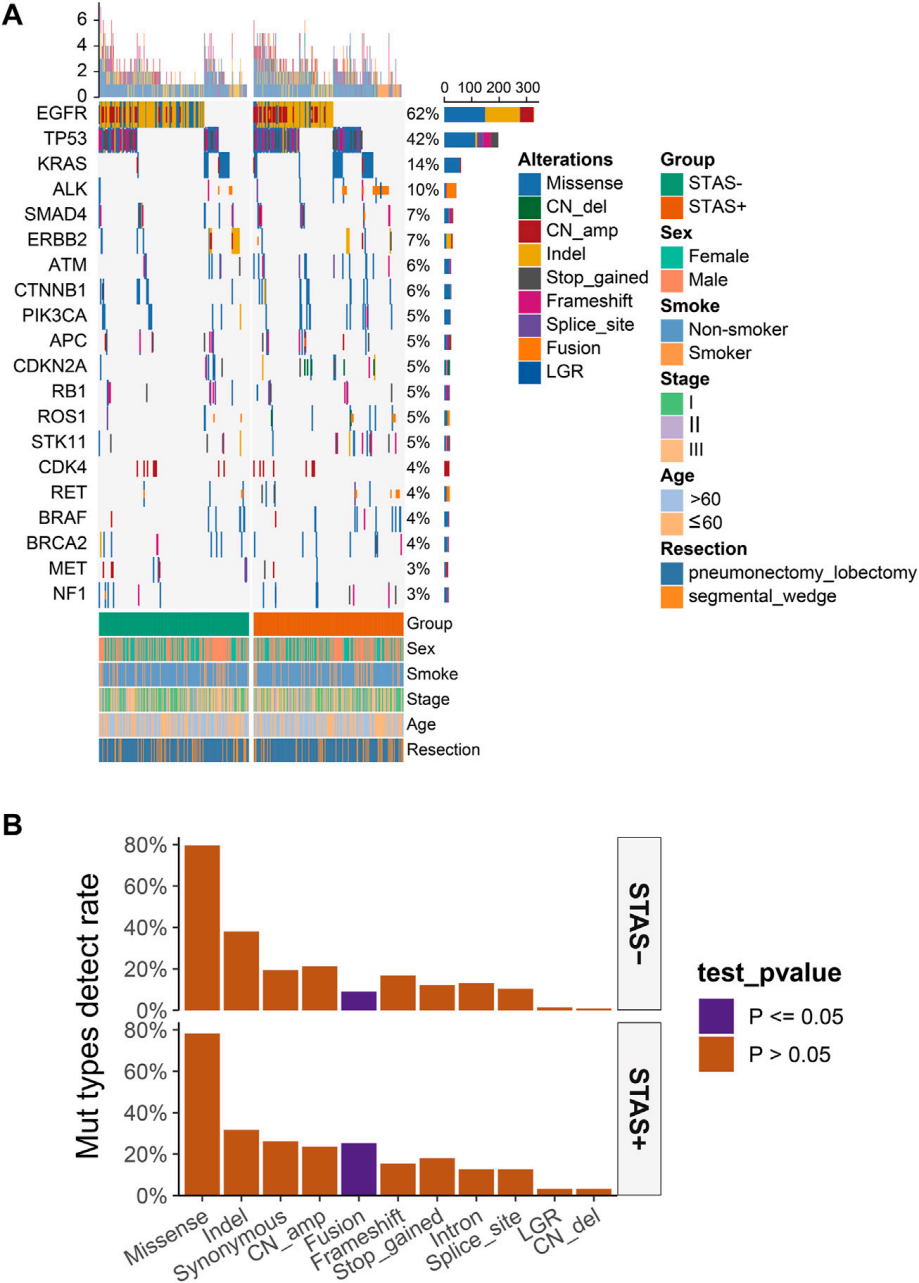
**(A)** Mutational spectrum of lung adenocarcinoma patients grouped according to STAS presence or absence. Gender, smoking status, age, disease stage, age, and type of surgery are also annotated at the bottom of the printout. Each column represents a patient, and each row represents a gene. The left column shows the mutated genes. The right column shows the mutation rate and the number of mutation types for each gene. The top plot represents the total number of mutations carried by the patient. Different colors indicate different types of mutations. **(B)** Overall lung adenocarcinoma patients were grouped according to STAS presence or absence for mutation type detection rates. The X-axis represents the mutation type, and the Y-axis represents the mutation frequency. STAS, propagation through the air space.

A total of 442 surgically resected ADC tissues were analyzed, of which 440 samples (99.6%) were positive for the overall mutational spectrum. The top six highest mutational genes were EGFR, TP53, KRAS, ALK, SMAD4, and ERBB2 (62%, 42%, 14%, 10%, 7%, and 7%, respectively). The most frequently detected mutation type was the missense mutation, with detection rates of 78.3% and 79.6% in SP and SN populations, respectively (*p* = 0.82). The SP population presented with a significantly high detection rate of fusion rearrangement types compared with the SN population (25.3% vs 9.0%, *p* < 0.05), and the remaining mutation types were not significantly different between the two groups (Figure 1).

### Characterization of somatic mutations

**FIGURE 2:**
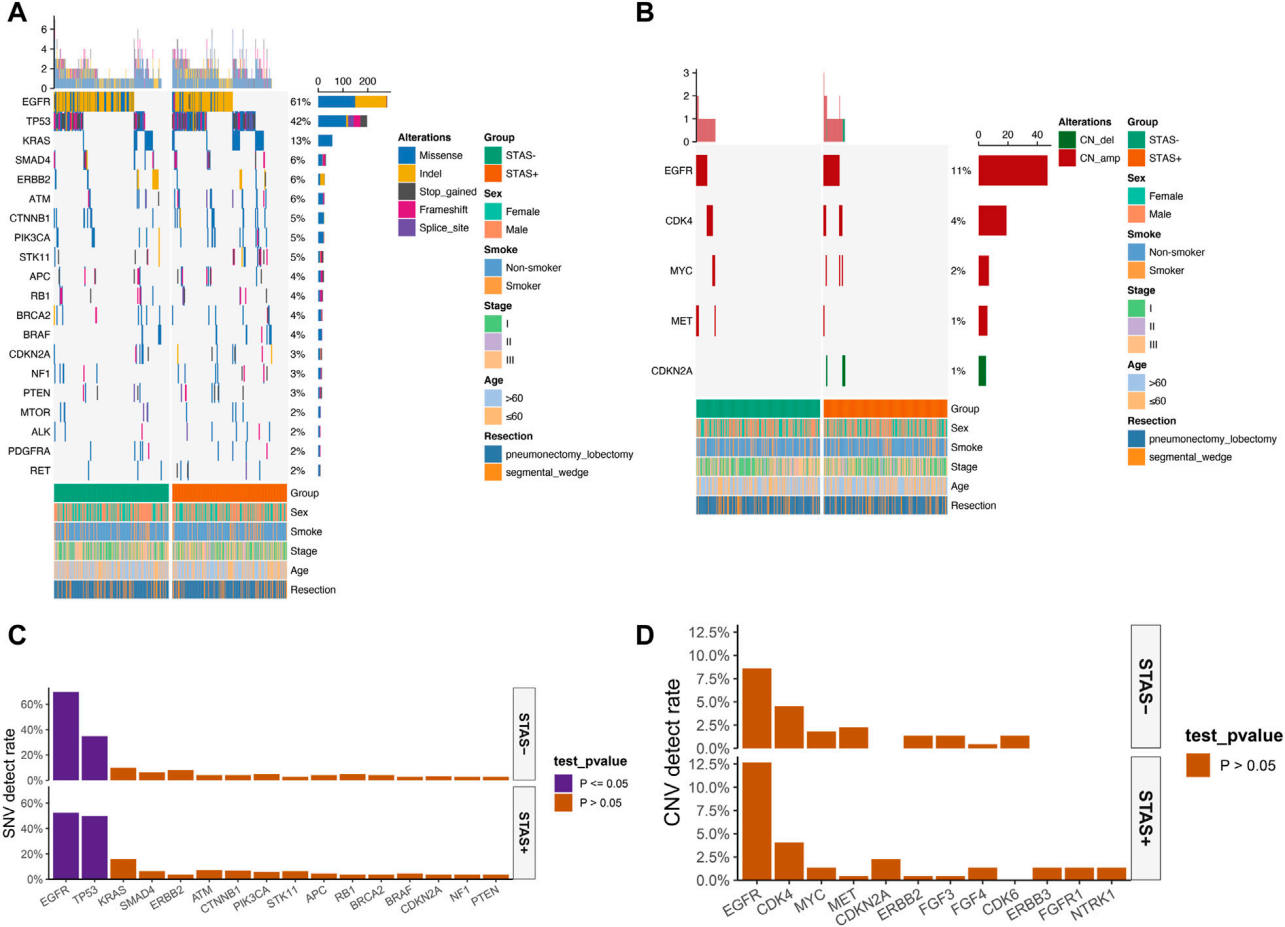
**(A)** Mutational spectrum of SNV of lung adenocarcinoma patients grouped according to STAS presence or absence. **(B)** Mutational spectrum of CNV of lung adenocarcinoma patients grouped according to STAS presence or absence. Gender, smoking status, disease stage, age, and type of surgery are annotated at the bottom of the printout. Each column represents a patient, and each row represents a gene. The left column shows the mutated genes. The right column shows the mutation rate and the number of mutation types for each gene. The top plot represents the total number of mutations carried by the patient. Different colors indicate different types of mutations. **(C)** SNV detection rate of mutation genes in overall lung adenocarcinoma patients grouped according to STAS presence or absence. **(D)** CNV detection rate of mutation genes in overall lung adenocarcinoma patients grouped according to STAS presence or absence. The X-axis represents the mutated genes, and the Y-axis represents the mutation frequency.

The detection rate of EGFR in the SP population in the single-nucleotide variant (SNV) mutation spectrum was significantly lower than that in the SN population (52.5% vs 69.7%, *p* < 0.05). The detection rate of TP53 in the SP population was significantly higher than that in the SN population (49.8% vs 34.8%, *p* < 0.05) (Figures 2A, C). No significant difference in copy number variation (CNV) of each gene was observed between the SP and SN populations in the CNV mutation profile (Figures 2B, D). The somatic mutations detected above 1% are demonstrated in Supplementary Figure S1.

### Characterization of mutational hotspots of driver genes

**FIGURE 3:**
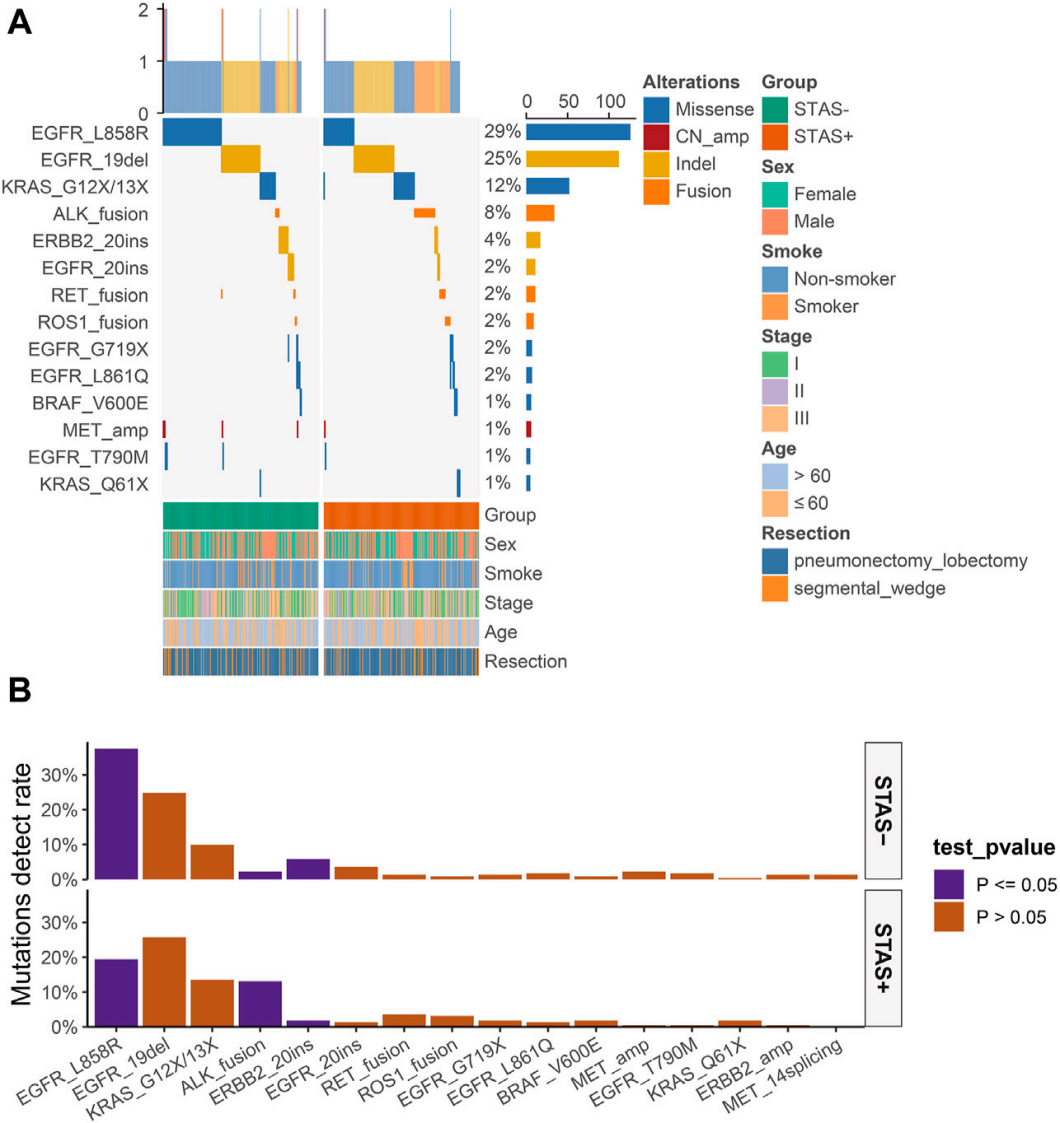
**(A)** Mutational hotspot spectrum of derived genes of lung adenocarcinoma patients grouped according to STAS presence or absence. Gender, smoking status, disease stage, age, and type of surgery are also annotated at the bottom of the printout. Each column represents a patient, and each row represents a gene. The left column shows the mutational hotspots of the derived genes. The right column shows the mutation rate of each gene and the number of mutation types for each gene. The top plot represents the total number of mutations carried by the patient. Different colors indicate different types of mutations. **(B)** Detection rate of mutational hotspots of derived genes in overall lung adenocarcinoma patients grouped according to STAS presence or absence. The X-axis represents the mutation hotspots of the derived genes, and the Y-axis represents the mutation frequency. STAS, transmission through the air space.

The detection rate of EGFR L858R missense mutation (19.5% vs 37.6%, *p* < 0.001) and ERBB2 exon 20 indel mutation (1.8% vs 5.9%, *p* < 0.05) was significantly lower in ADC mutational hotspots of driver genes in the SP population. The detection rate of ALK fusion rearrangements in the SP population was significantly higher than that in the SN population (13.1% vs 2.3%, *p* < 0.05) (Figure 3).

### Signaling pathway analysis

To investigate the potential tumor-related signaling pathway differences in SP and SN populations, we calculated the mutation detection rates of genes in some tumor-related signaling pathways. In this part, a significant difference in gene mutation primarily existed in the P53 cell cycle signaling pathway and Wnt and ERBB signaling pathways (Supplementary Figure S2A).

In the P53 cell cycle signaling pathway, we observed significantly more TP53 mutations in SP patients than in SN patients (49.8% vs 34.8%, *p* < 0.01). Moreover, the mutation detection rates of other P53 cell cycle signaling pathway-related genes, including ATM, CDKN2A, and TSC2, were also slightly higher in the SP population than in the SN population (7.2% vs 4.1%, 5.9% vs 3.6%, and 4.1% vs 1.4%. *p* = 0.22, 0.37, and 0.14, respectively) (Supplementary Figure S2B). For the Wnt signaling pathway, TP53 mutations showed a dominant difference between SP and SN populations (49.8% vs 34.8%, *p* < 0.01). In addition, the mutation detection rates of SMAD4 and CTNNB1 in SP patients were slightly higher than those in SN patients (7.2% vs 6.3% and 6.8% vs 4.5%. *p* = 0.85 and 0.41, respectively) (Supplementary Figure S2C). However, the mutation rate of the ERBB signaling pathway-related genes was lower in the SP population, including EGFR and ERBB2 mutations (52.9% vs 70.1%, *p* < 0.01; 4.5% vs 8.6%, *p* = 0.12) (Supplementary Figure S2D). The detection rates for each gene in the signaling pathway are shown in Supplementary Tables S2A–S2C.

### Exclusivity and co-occurrence of mutations and NMF cluster analysis

**FIGURE 4:**
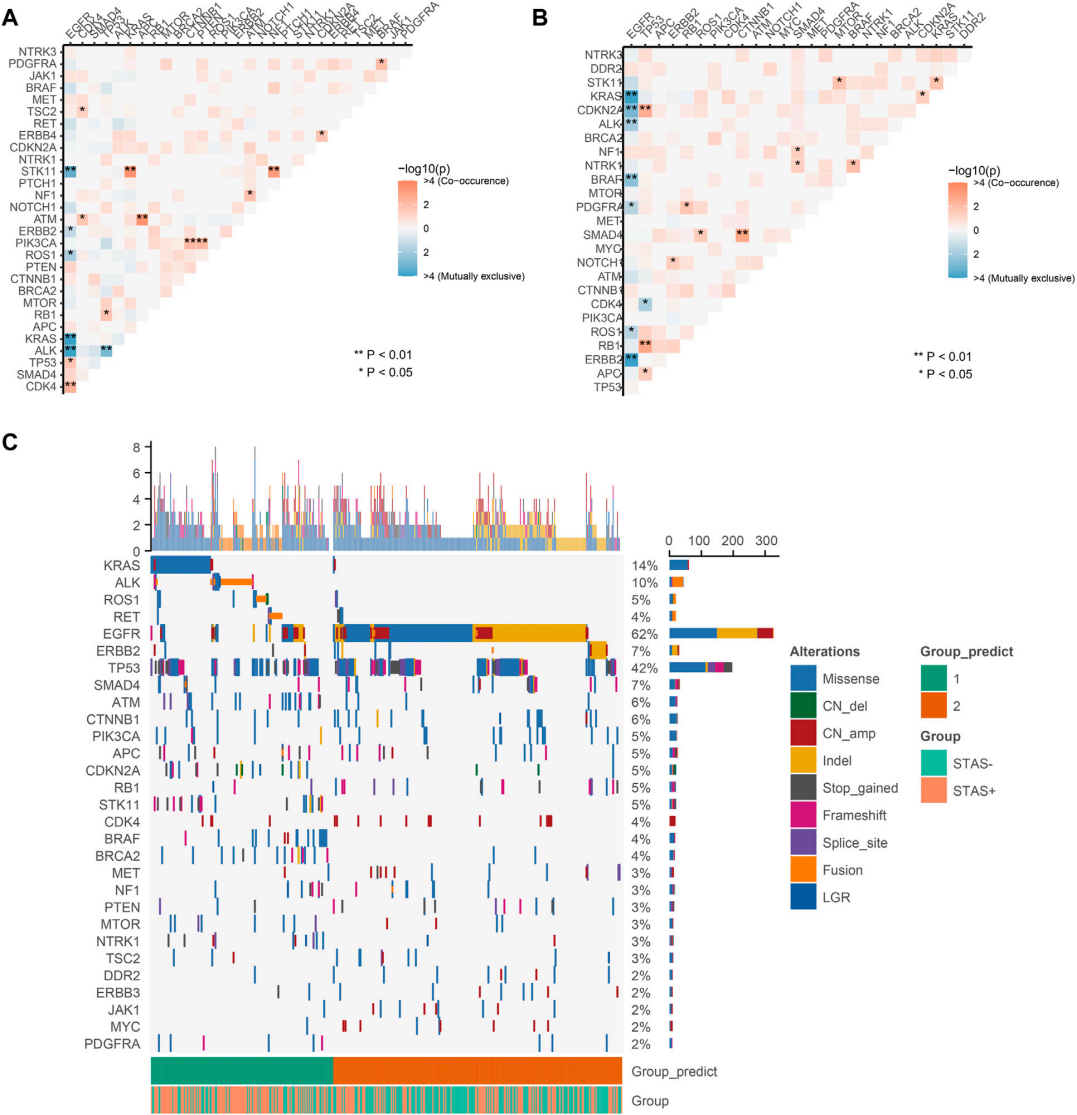
**(A)** Pairwise assessment of mutual exclusivity and association in driver genes of the STAS-positive group. Orange–red is associated with positive correlations, while blue indicates mutual repulsion. Asterisks indicate important relationships. **p < 0.01, *p < 0.05. **(B)** Pairwise assessment of mutual exclusivity and association in driver genes of the SN group. Orange–red is associated with positive correlations, while blue indicates mutual repulsion. Asterisks indicate important relationships. **p < 0.01, *p < 0.05. **(C)** NMF clustering of all genes based on somatic mutations from 442 lung adenocarcinoma patients. Hierarchical clustering revealed that in cluster 1, there were 171 samples, of which 111 (64.9%) were SP consisting of the majority of KRAS, ALK, STK11, ROS1, ATM, and RET mutations, and in cluster 2, there were 271 samples, of which 161 (59.4%) were SN consisting of the majority of EGFR, ERBB2, SMAD4, and CTNNB1 mutations. Each column represents a patient, and each row represents a gene. The left column shows the mutated genes. The right column shows the mutation rate and the number of mutation types for each gene. The top plot represents the total number of mutations carried by the patient. Different colors indicate different types of mutations. STAS, propagation through the air space.

The results of exclusivity and co-occurrence analyses were examined in this part. In SP subjects, EGFR and driver genes, such as ALK, KRAS, ROS1, and ERBB2, were mutually exclusive; EGFR was previously mutually exclusive with STK11. PIK3CA alterations were substantially correlated with aberrations of CTNNB1 and PTEN; KRAS and STK11 co-alterations occurred frequently (Figure 4A). Among SN subjects, EGFR was mutually exclusive with driver genes, such as ALK, KRAS, ROS1, ERBB2, and BRAF. We detected TP53 and CDKN2A/RB1/APC co-alterations, and SMAD4 alterations correlated with aberrations of CTNNB1. In addition, mutations in KRAS and STK11 co-occurred in the SN population (*p* < 0.05) with less significance than that in SP subjects (*p* < 0.001) (Figure 4B).

The numerical matrix of all samples and their gene mutations was iteratively reduced by non-negative matrix factorization (NMF) and divided into two categories. There were 171 samples in cluster 1, of which 111 (64.9%) were SP. The clustering features showed the occurrence of KRAS, ALK, STK11, ROS1, ATM, and RET mutations. There were 271 samples in cluster 2, of which 161 (59.4%) were SN. The clustering features showed the occurrence of mutations in EGFR, ERBB2, SMAD4, and CTNNB1. Figure 4C presents STAS-positive and -negative subgroups that had similar contributions in the two groups predicted by unsupervised clustering. In addition, a higher proportion of STAS-positive patients were observed in the two predicted groups. In the NMF results, it was difficult to classify STAS-positive and -negative populations only by mutation spectrum characteristics.

### Postoperative follow-up

**FIGURE 5:**
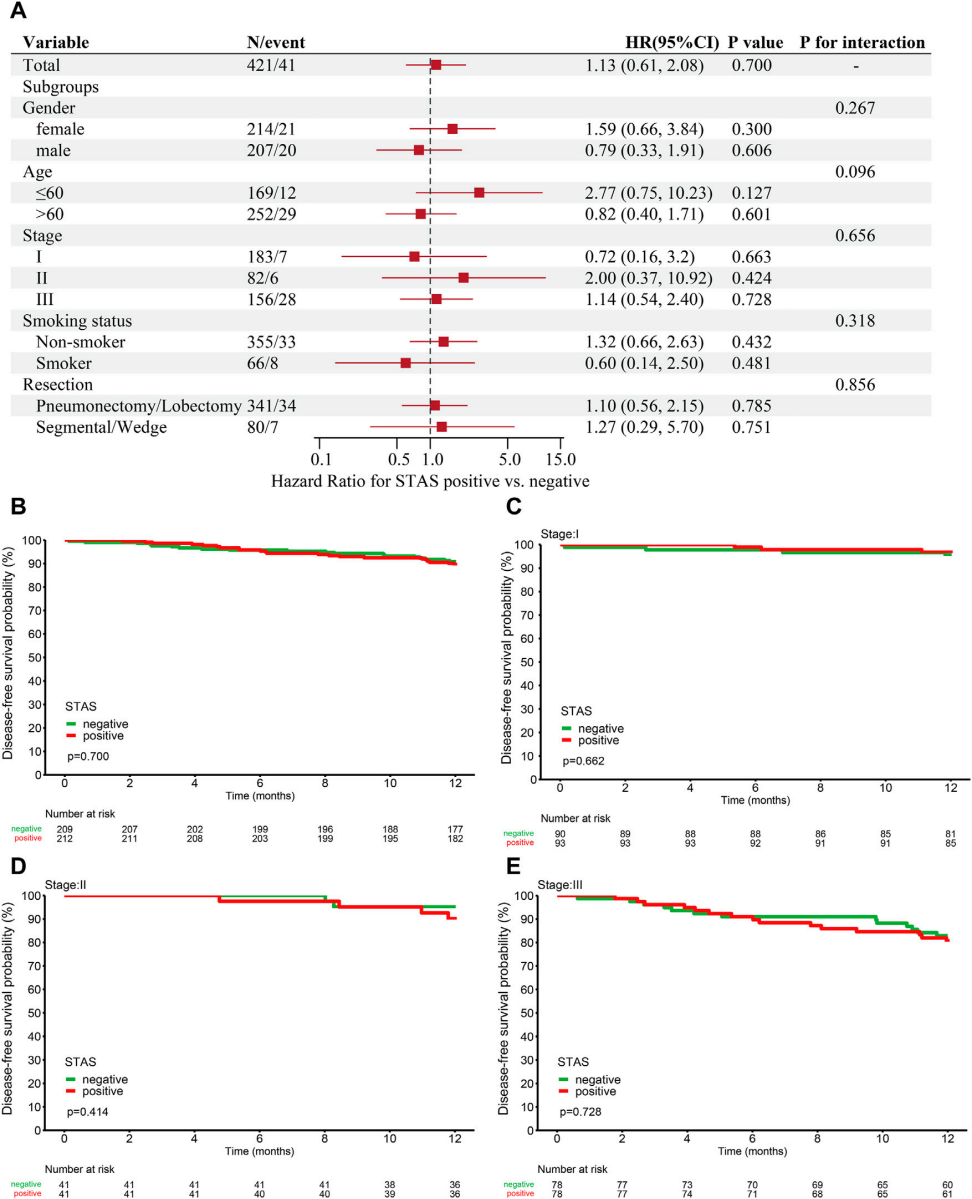
**(A)** Analysis of the relationship between prognosis and clinical characteristics in STAS-positive and -negative groups. One-year disease-free survival (DFS) for this cohort. **(B)** DFS based on overall patients grouped according to STAS presence or absence. **(C)** DFS based on stage I patients. **(D)** DFS based on stage II patients. **(E)** DFS based on stage III patients. STAS, propagation through the air space; HR, hazard ratio; CI, confidence interval.

All patients were regularly followed up every 3 months for a year. One subject died during surgery, and 20 subjects were lost to follow-up. The final follow-up date was 28 February 2022. We tested the correlation between STAS and prognosis in 421 patients with fully resected stage I–III ADC. Additionally, the results showed no significant link between clinical characteristics and prognosis in either group. A total of 421 subjects were included for further prognostic analyses. A total of 41 cases (9.7%) relapsed or died within 1 year, including 22 SP cases (10.4%) and 19 SN cases (9.1%). The association between tumor stage and prognosis in these patients were also investigated [stage I (HR, 0.72, *p* = 0.663), stage II (HR, 2.00, *p* = 0.424), and stage III (HR, 1.14, *p* = 0.728)]. The results are presented in Figure 5A.

The 1-year DFS in the STAS-positive or -negative group of all ADC patients was 89.6% and 90.7% (*p* = 0.7, Figure 5B), respectively. The 1-year DFS in the STAS^+/−^subgroup of patients with stage I lung cancer was 96.8% and 95.5% (*p* = 0.662, Figure 5C), the 1-year DFS in the STAS^+/−^subgroup of patients with stage II lung cancer was 90.1% and 95.1% (*p* = 0.414, Figure 5D), and the STAS^+/−^subgroup of patients with stage III lung cancer had a 1-year DFS of 80.7% and 82.9% (*p* = 0.728, Figure 5E), respectively. The 1-year DFS of the SP population did not differ from that of the SN patients in this study.

## Discussion

STAS is known as an indicator of poor prognosis for lung cancer and is associated with a high possibility of early local recurrence and segmentation. However, the molecular characteristics and the mechanisms of association of STAS patients remain incompletely understood. With this in mind, we compared the genetic profiles of lung adenocarcinoma patients with and without STAS to elucidate the mutation profiles and biological relationships associated with STAS patients.

In this study, we found that the fusion rearrangement mutation detection rate is significantly higher in the SP population (25.3% in STAS^+^ and 9.0% in STAS^−^). EGFR mutation had significantly lower detection rates in the SP population than in the SN population (52.5% in STAS^+^ and 69.7% in STAS^−^). We also found that EGFR L858R (19.5% in STAS^+^ and 37.6% in STAS^−^) and ERBB2 20ins (1.8% in STAS^+^ and 5.9% in STAS^−^) of mutational hotspots of derived genes in the SP group were dramatically lower than those in the SN group. The gene with a significantly higher detection rate in the SP population than in the SN population was TP53 (49.8% in STAS^+^ and 34.8% in STAS^−^). In the SP group, the mutated hotspot of derived genes was ALK fusion (13.1% in STAS^+^ and 2.3% in STAS^−^). Other gene mutations, such as KRAS, ROS1, and BRAF, were not differently expressed between the SP and SN populations. Similarly, several studies have demonstrated that STAS is correlated with a low mutation rate of EGFR (Warth et al., 2015; Lee et al., 2018; Jia et al., 2020). At the same time, STAS was also found to be more frequently observed in ALK-rearranged lung cancer (Lee et al., 2018; Kadota et al., 2019; Jia et al., 2020). In addition, Tian et al. (2021) found that the occurrence of STAS is often accompanied by the increased expression of TP53. Importantly, most studies also found that KRAS (Warth et al., 2015; Lee et al., 2018; Zeng et al., 2020) is not related to STAS, which is proved in the current study. However, some studies propose a totally opposite conclusion. Several research studies suggest that there is no correlation between STAS and EGFR (Toyokawa et al., 2018a; Toyokawa et al., 2018c; Zhang Z. et al., 2020). Meanwhile, two other studies reported that high mutation rates of ROS1 (Jia et al., 2020) and BRAF (Warth et al., 2015) were associated with the occurrence of STAS. Ethnic diversity and different testing approaches can cause contradictions.

We also explored the gene mutations in some important tumor-related signaling pathways in SP and SN populations, including the P53 cell cycle signaling pathway and Wnt and ERBB signaling pathways. It is well known that genomic rearrangements associated with ERBB networks represent one of the major driver mutations and potential targets for establishing novel ADC treatment modalities (Trombetta et al., 2017; Kruspig et al., 2018). Many studies have shown that, as important genes constituting the ERBB network, EGFR (da Cunha Santos et al., 2011) and ERBB2 (20ins) (Xiang et al., 2022) are significantly related to the development and invasion of lung cancer. At the same time, EGFR has also been found to be closely related to STAS (Warth et al., 2015; Lee et al., 2018). Similarly, we have observed a lower detection rate of EGFR and ERBB2 (20ins) in the SP population, which is the major discovery in the ERBB signaling pathway. In addition, TP53 is a major tumor suppressor gene and the most frequently inactivated gene in cancer and plays an important role in both TP53 cell cycle signaling and Wnt signaling pathways. Prevention and suppression of TP53 mutation are one of the approaches to be considered in the treatment of tumors (Bykov et al., 2018). In this study, we have discovered a higher TP53 mutation (missense) rate in the SP population in the analysis of not only the P53 cell cycle signaling pathway but also the Wnt signaling pathway. Similarly, TP53 has been previously proved to be a common alteration in SP adenocarcinomas (Cardona et al., 2018). However, we cannot deduce that the P53 cell cycle signaling pathway and Wnt signaling pathway are different between SP and SN patients according to the results of TP53 mutation, as only a minority of these signaling pathway-related genes have been detected in this research. Collectively, the alteration of all p53 cell cycle, Wnt, and ERBB signaling pathway-related genes is not completely understood in this article. Therefore, more studies are needed to better understand STAS.

Furthermore, we analyzed the mutual exclusivity and co-occurrence of genes in both groups and found that in the SP population, EGFR excluding driver genes, including ALK, KRAS, ROS1, ERBB2, EGFR, and STK11, was mutually exclusive, while PIK3CA co-occurred with CTNNB1/PTEN, and KRAS co-occurred with STK11. In the SN population, EGFR and driver genes, including ALK, KRAS, ROS1, ERBB2, and BRAF, were mutually exclusive. TP53 co-occurred with CDKN2A, RB1, and APC, and SMAD4 co-occurred with CTNNB1. It is known that analysis of mutually exclusive co-occurrence of genes aids the exploration of the pathogenic pathway of tumors and provides essential references for the localization of driver genes (Sanchez-Vega et al., 2018; Scheffler et al., 2019). Previous large-scale genome sequencing data have indicated that mutations driving oncogenes, such as KRAS *versus* EGFR, are often mutually exclusive (Gainor et al., 2013), which is consistent with our results. Interestingly, Cisowski and Bergo (2017) suggested in a mouse lung cancer model that the co-occurrence of two tumor driver genes (KRAS and BRAF) is deleterious, leading to cell cycle exit, senescence, and death. It is reported that mutations in STK11 can be found in 19.8% of KRAS-mutated NSCLCs, which is associated with poor overall survival (Facchinetti et al., 2017; Scheffler et al., 2019). Based on our current results, STAS may detrimentally effect patients’ overall survival as co-alterations of KRAS and STK11 occur more frequently in SP subjects. Alhough a previous study has proved that alterations in the PI3K pathway most likely occur as isolated events in lung cancer (Millis et al., 2019), the mutations of PTEN and PIK3CA, both PI3K pathway-related genes, also co-occur frequently in the SP population. However, the role of the co-occurrence of PTEN and PIK3CA in STAS is not clear yet.

We found there are differences between the two groups in terms of mutation, so we tried to use unsupervised clustering and dimensionality reduction to discover whether the overall mutation characteristics of different groups of STAS could be extracted. Unfortunately, mutation characteristics are affected by many factors, and unsupervised clustering could not accurately extract the mutation characteristics of the two groups of STAS. The reason why STAS cannot be distinguished is that, on the one hand, lung adenocarcinoma is a well-defined driving type, and the molecular typing induced by driving is more significant than the STAS classification. On the other hand, the NMF model has been used in the application of mutation spectrum dimensionality reduction *via* limited numerical transformation (Jiang et al., 2022).

Numerous studies have revealed that STAS is an independent prognostic indicator for poor outcomes (Kadota et al., 2015; Shiono and Yanagawa, 2016; Toyokawa et al., 2018d; Zhang B. et al., 2020; Ding et al., 2020). The 1-year DFS did not differ significantly between STAS-positive and -negative patients in our study. Because of the relatively short follow-up period, the outcome measures were limited. We only analyzed the 1-year DFS of these subjects in this study. Further multicentric prospective studies are required to provide more convincing results, especially studies analyzing the prognostic effects of genomic alterations when tumor spread through the air space is taken into account.

The limitations of our study were as follows: first, the relatively small sample size and the small size of the NGS panel limited the analysis on less common genomic alterations and STAS associations. Second, the PSM approach has its intrinsic limitations. For instance, there may have been other clinical features that were not included in this regression model.

## Conclusion

In summary, the present study analyzed the genomic characteristics of STAS-positive and -negative populations in a relatively large population cohort of lung adenocarcinoma patients. It demonstrated the global genetic mutation profiles of STAS-positive and -negative lung adenocarcinoma patients.

## Data Availability

The original contributions presented in the study are publicly available. This data can be found here: NGDC. CNCB, PRJCA013869

## Supplementary material

SUPPLEMENTARY FIGURE S1
**(A)** Gene mutations detected greater than 1% in the STAS-positive patients. **(B)** Gene mutations detected greater than 1% in the STAS-negative patients. STAS, propagation through the air space.

SUPPLEMENTARY FIGURE S2
**(A)** Detection rate of signaling pathways in overall lung adenocarcinoma patients grouped according to STAS presence or absence. The X-axis represents the signaling pathway names, and the Y-axis indicates the detection frequency. **(B)** Mutational spectrum of the P53 cell cycle signaling pathway of lung adenocarcinoma patients grouped according to STAS presence or absence. **(C)** Mutational spectrum of the Wnt signaling pathway of lung adenocarcinoma patients grouped according to STAS presence or absence. **(D)** Mutational spectrum of the ERBB signaling pathway of lung adenocarcinoma patients grouped according to STAS presence or absence. Gender, smoking status, disease stage, age, and type of surgery are annotated at the bottom of the printout. Each column represents a patient, and each row represents a gene. The left column shows the mutated genes. The right column shows the mutation rate of each gene and the number of mutation types for each gene. The top plot represents the total number of mutations carried by the patient. Different colors indicate different types of mutations.
