# Supplementary material for: Comprehensive molecular characterizations of stage I–III lung adenocarcinoma with tumor spread through air spaces
Source: Front Genet. 2023 Feb 2;14:1101443. doi: 10.3389/fgene.2023.1101443 (PMC9932204; doi:10.3389/fgene.2023.1101443)
Supplement: Supplementary file 1 [file Table1.DOCX]

**Supplemental Table 1.** OncoScreen 68 genes list

ALK BRAF EGFR ERBB2 KRAS MET RET ROS1 AKT1 APC AR ARAF ATM AXL BCL2L11 BRCA1 BRCA2 CCND1 CD74 CDK4 CDK6 CDKN2A CTNNB1 DDR2 ERBB3 ESR1 ERBB4 FGF19 FGF3 FGF4 FGFR1 FGFR2 FGFR3 FLT3 HRAS IDH1 IDH2 IFG1R JAK1 JAK2 KDR KIT MAP2K1 MTOR MYC NF1 NOTCH1 NRAS NRG1 NTRK1 NTRK2 NTRK3 PDGFRA PIK3CA PTCH1 PTEN RAF1 RB1 SMAD4 SMO STK11 TOP2A TP53 TSC1

TSC2 CYP2D6 DPYD UGT1A1
