# Supplementary figures and images for: Comprehensive molecular characterizations of stage I–III lung adenocarcinoma with tumor spread through air spaces
Source: Front Genet. 2023 Feb 2;14:1101443. doi: 10.3389/fgene.2023.1101443 (PMC9932204; doi:10.3389/fgene.2023.1101443)

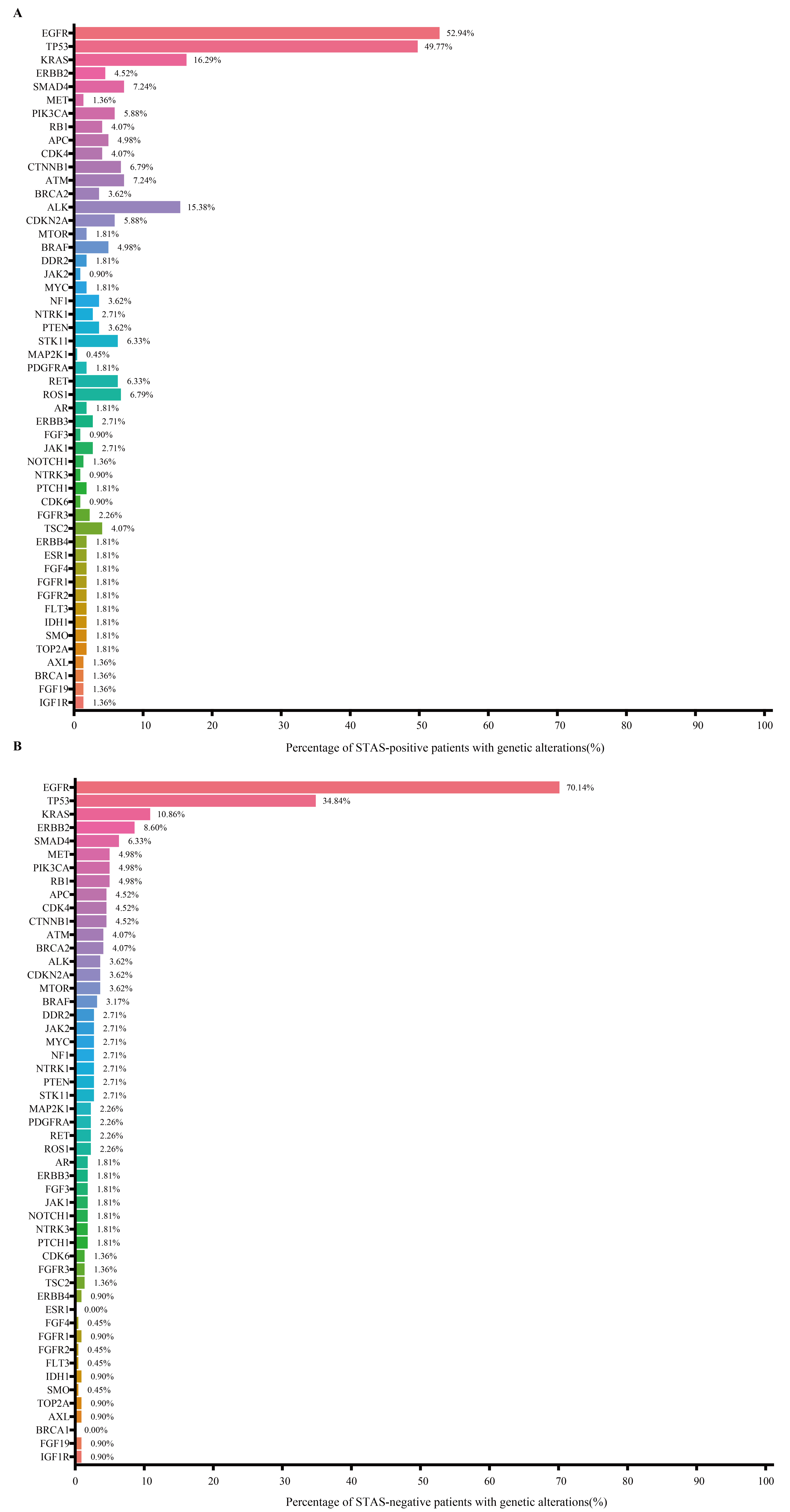

Supplement: Supplementary file 2 [file Image1.JPEG]
