# Supplementary material for: Comprehensive molecular characterizations of stage I–III lung adenocarcinoma with tumor spread through air spaces
Source: Front Genet. 2023 Feb 2;14:1101443. doi: 10.3389/fgene.2023.1101443 (PMC9932204; doi:10.3389/fgene.2023.1101443)
Supplement: Supplementary file 4 [file Table2.DOCX]

| **Supplemental Table 2A.**The detection rates for each gene in the P53 pathway | | | | |
| --- | --- | --- | --- | --- |
| gene | Group | detect_n | cohort_n | detect_rate |
| TP53 | STAS+ | 110 | 221 | 0.49773756 |
| TP53 | STAS- | 77 | 221 | 0.34841629 |
| ATM | STAS+ | 16 | 221 | 0.07239819 |
| CDKN2A | STAS+ | 13 | 221 | 0.05882353 |
| CDK4 | STAS- | 10 | 221 | 0.04524887 |
| ATM | STAS- | 9 | 221 | 0.04072398 |
| CDK4 | STAS+ | 9 | 221 | 0.04072398 |
| TSC2 | STAS+ | 9 | 221 | 0.04072398 |
| CDKN2A | STAS- | 8 | 221 | 0.0361991 |
| PTEN | STAS+ | 8 | 221 | 0.0361991 |
| PTEN | STAS- | 6 | 221 | 0.02714932 |
| CDK6 | STAS- | 3 | 221 | 0.01357466 |
| TSC2 | STAS- | 3 | 221 | 0.01357466 |
| CCND1 | STAS- | 2 | 221 | 0.00904977 |
| CCND1 | STAS+ | 2 | 221 | 0.00904977 |
| CDK6 | STAS+ | 2 | 221 | 0.00904977 |

| **Supplemental Table 2B.**The detection rates for each gene in the Wnt pathway | | | | |
| --- | --- | --- | --- | --- |
| gene | Group | detect_n | cohort_n | detect_rate |
| TP53 | STAS+ | 110 | 221 | 0.49773756 |
| TP53 | STAS- | 77 | 221 | 0.34841629 |
| SMAD4 | STAS+ | 16 | 221 | 0.07239819 |
| CTNNB1 | STAS+ | 15 | 221 | 0.0678733 |
| SMAD4 | STAS- | 14 | 221 | 0.06334842 |
| APC | STAS+ | 11 | 221 | 0.04977376 |
| APC | STAS- | 10 | 221 | 0.04524887 |
| CTNNB1 | STAS- | 10 | 221 | 0.04524887 |
| MYC | STAS- | 6 | 221 | 0.02714932 |
| MYC | STAS+ | 4 | 221 | 0.01809955 |
| CCND1 | STAS- | 2 | 221 | 0.00904977 |
| CCND1 | STAS+ | 2 | 221 | 0.00904977 |

| **Supplemental Table 2C.**The detection rates for each gene in the ERBB pathway | | | | |
| --- | --- | --- | --- | --- |
| gene | Group | detect_n | cohort_n | detect_rate |
| EGFR | STAS- | 155 | 221 | 0.70135747 |
| EGFR | STAS+ | 117 | 221 | 0.52941176 |
| KRAS | STAS+ | 36 | 221 | 0.16289593 |
| KRAS | STAS- | 24 | 221 | 0.10859729 |
| ERBB2 | STAS- | 19 | 221 | 0.08597285 |
| PIK3CA | STAS+ | 13 | 221 | 0.05882353 |
| BRAF | STAS+ | 11 | 221 | 0.04977376 |
| PIK3CA | STAS- | 11 | 221 | 0.04977376 |
| ERBB2 | STAS+ | 10 | 221 | 0.04524887 |
| MTOR | STAS- | 8 | 221 | 0.0361991 |
| BRAF | STAS- | 7 | 221 | 0.03167421 |
| ERBB3 | STAS+ | 6 | 221 | 0.02714932 |
| MYC | STAS- | 6 | 221 | 0.02714932 |
| MAP2K1 | STAS- | 5 | 221 | 0.02262443 |
| ERBB3 | STAS- | 4 | 221 | 0.01809955 |
| ERBB4 | STAS+ | 4 | 221 | 0.01809955 |
| MTOR | STAS+ | 4 | 221 | 0.01809955 |
| MYC | STAS+ | 4 | 221 | 0.01809955 |
| AKT1 | STAS+ | 2 | 221 | 0.00904977 |
| ARAF | STAS+ | 2 | 221 | 0.00904977 |
| ERBB4 | STAS- | 2 | 221 | 0.00904977 |
| HRAS | STAS- | 1 | 221 | 0.00452489 |
| MAP2K1 | STAS+ | 1 | 221 | 0.00452489 |
| NRG1 | STAS+ | 1 | 221 | 0.00452489 |
| RAF1 | STAS- | 1 | 221 | 0.00452489 |
| RAF1 | STAS+ | 1 | 221 | 0.00452489 |
